# Perinatal death beyond 41 weeks pregnancy: an evaluation of causes and substandard care factors as identified in perinatal audit in the Netherlands

**DOI:** 10.1186/s12884-018-1973-0

**Published:** 2018-09-20

**Authors:** Joep C. Kortekaas, Anke C. Scheuer, Esteriek de Miranda, Aimée E. van Dijk, Judit K. J. Keulen, Aafke Bruinsma, Ben W. J. Mol, Frank P. H. A. Vandenbussche, Jeroen van Dillen

**Affiliations:** 1 Department of Obstetrics and Gynaecology, Radboud University Medical Center, Geert Grooteplein Zuid 10, 6523 GA Nijmegen, the Netherlands; 2 Department of Obstetrics and Gynaecology, Amsterdam University Medical Centers, Amsterdam, the Netherlands; 3 Perined, Utrecht, the Netherlands; 4 Department of Obstetrics and Gynaecology, Monash University, Clayton, VIC Australia

**Keywords:** CTG, Delivery, Foetal monitoring, Late-term pregnancy, Mortality, Postterm pregnancy, Pregnancy, Stillbirth

## Abstract

**Background:**

Late- and postterm pregnancy are associated with adverse perinatal outcomes, like perinatal death. We evaluated causes of death and substandard care factors (SSFs) in term and postterm perinatal death.

**Methods:**

We used data from the Perinatal Audit Registry of the Netherlands (PARS). Women with a term perinatal death registered in PARS were stratified by gestational age into early−/full-term (37.0–40.6) and late−/postterm (≥41.0 weeks) death. Cause of death and SSFs ≥41 weeks were scored and classified by the local perinatal audit teams.

**Results:**

During 2010–2012, 947/479,097 (0.21%) term deaths occurred, from which 707 cases (75%) were registered and could be used for analyses. Five hundred ninety-eight early−/full-term and 109 late−/postterm audited deaths were registered in the PARS database. Of all audited cases of perinatal death in the PARS database, 55.2% in the early-/fullterm group occurred antepartum compared to 42.2% in the late−/postterm group, while intrapartum death occurred in 7.2% in the early−/full-term group compared to 19.3% in the late−/postterm group in the audited cases from the PARS database. According to the local perinatal audit, the most relevant causes of perinatal death ≥41 weeks were antepartum asphyxia (7.3%), intrapartum asphyxia (9.2%), neonatal asphyxia (10.1%) and placental insufficiency (10.1%). In the group with perinatal death ≥41 weeks there was ≥1SSF identified in 68.8%. The most frequent SSFs concerned inadequate cardiotocography (CTG) evaluation and/or classification (10.1%), incomplete registration or documentation in medical files (4.6%) or inadequate action on decreased foetal movements (4.6%).

**Conclusions:**

In the Netherlands Perinatal Audit Registry, stillbirth occurred relatively less often antepartum and more often intrapartum in pregnancies ≥41 weeks compared to pregnancies at 37.0–40.6 weeks in the audited cases from the PARS database. Foetal, intrapartum and neonatal asphyxia were identified more frequently as cause of death in pregnancies ≥41 weeks. The most identified SSFs related to death in pregnancies ≥41 weeks concerned inadequate CTG monitoring (evaluation, classification, registration or documentation) and inadequate action on decreased foetal movements.

## Backgound

The incidence of postterm pregnancies (≥42.0 weeks) in European countries varies from 0.5% (Austria/Belgium) to 9% (Denmark/Sweden), depending on accurate pregnancy dating and clinical management protocols [1–5]. Ongoing pregnancy from 41 weeks onwards is associated with an increased risk of perinatal death and morbidity although the absolute risk of perinatal death is low [2, 6–8]. In the Perinatal Registry Netherlands (PRN), 534,058 births were registered between 2010 and 2012; 380,252 singletons were born between 37.0–40.6 weeks and 98,845 were born ≥41.0 weeks of gestation. The overall perinatal mortality rate in term and postterm singleton births per ongoing pregnancy in the gestational age interval was 0.20% (947/479,097). In the early−/full-term pregnancy (37.0–40.6) a perinatal mortality rate of 0.21% (787/380,252) was found and 0.16% (160/98,845) in pregnancies ≥41.0 weeks [9].

Controversy about the clinical management of an uncomplicated pregnancy reaching 41 weeks concerns the question whether labour should be induced at 41.0 weeks or if expectant management until 42.0 weeks could be allowed considering the prevention of adverse perinatal outcomes such as perinatal death [1, 4, 6, 10, 11]. In the Netherlands, this question has been identified by Dutch midwives and gynaecologists as the main dilemma in obstetrical policy in the term period resulting in the INDEX trial: a randomised clinical trial in which effects and costs of both induction of labour at 41.0 weeks and expectant management until 42.0 weeks are studied [12].

The aim of this perinatal audit study was to gain more insight into perinatal death in early−/full- (37.0–40.6 weeks) and late−/postterm (≥41.0 weeks) pregnancies by analysing the causes of death. A second goal of this study was to describe substandard care factors (SSFs) of all audited perinatal deaths at or beyond 41 weeks of gestation.

## Methods

A quantitative descriptive study was conducted on data from the Perinatal Audit Registry System (PARS) from 2010 to 2012. No ethical approval was needed according to the Dutch Central Committee of Human Research, because it concerns a study with anonymous data.

### Aim, design and setting of the study

All birth outcomes, including death, in pregnancies with a delivery of ≥22.0 weeks of gestation are anonymously entered in the Perinatal Registry Netherlands (PRN). A nationwide perinatal mortality audit was introduced in the Netherlands in 2010 by the foundation Perinatal Audit in the Netherlands (PAN) [9, 13]. Perined, a merger of PAN and the PRN, manages three web-based databases with anonymous (information not traceable to individual patients) registration on case level: the PRN, the Perinatal Audit Registry of the Netherlands (PRN-audit) and PARS. Cases of perinatal death are registered in the PRN audit. After registration, an anonymous narrative of each case is automatically constructed for use in the local perinatal audit. The aim to audit all cases of perinatal death was not always fulfilled. Based on content, some cases are selected by the local team to evaluate in the local perinatal audit. For example, cases can be chosen based on impact of the case on the obstetric caregivers involved, to stimulate modifications in local obstetric care to prevent future cases or because cases contain a rare event which can be informative to any obstetric caregiver. Cases are then evaluated by a multidisciplinary team, consisting of gynaecologists, midwives, paediatricians, general practitioners, and nurses, using the format of the local perinatal audit. In the local perinatal audit, the quality of perinatal care, the cause of the perinatal deaths, and the presence of SSFs are identified and systematically and critically analysed [14–16]. Time of death is determined by using the Wigglesworth/Hey classification [17, 18]. Cause of death is determined by using the modified relevant condition of death (ReCoDe). The modified ReCoDe was created by PAN and contains the ReCoDe of Gardosi added with the neonatal classification of Chan and maternal risk factors [19, 20]. On each case, more than one clinical condition can be chosen as cause of death and in each case one item with the ‘most important relation to death’ is chosen. A detailed handbook for definition and classification was distributed to each local perinatal audit team [21]. The results of the audit process are registered in PARS by a trained representative of each local perinatal audit team [17–19, 22, 23]. Data used for this study originated from the registered cases of perinatal death in PARS. Details about the organisation and training of care providers participating in the Perinatal Audit is described by Eskes [23].

### Characteristics of participants

Based on the Wigglesworth/Hey classification (WHEN) all perinatal deaths of ≥37 weeks in the PARS registry could be selected. A comparison could be made between early−/full-term pregnancy (37.0–40.6 weeks of pregnancy) and late−/postterm pregnancy (≥41 weeks). Unfortunately, discrimination between the late-term and postterm group was not possible in the PARS registry, only a group ≥41 weeks could be selected.

### Description of processes, interventions and comparisons

A handbook for definition and classification of perinatal death was distributed to each local perinatal audit team. Stillbirth is defined as all antepartum and intrapartum stillbirth combined. Neonatal death is specified as all deaths after livebirth until 28 days and is divided in early (day 0–6) and late (day 7–27). Foetal asphyxia is defined as pathological changes caused by lack of oxygen, resulting in hypoxia and hypercapnia. Intrapartum asphyxia is defined as evidence of severe hypoxemia after the onset of labour, in the presence of contractions [21]. To determine foetal well-being before or during labour, foetal cardiac rhythm is monitored either by doptone (low risk pregnancies in midwifery-led care) or by cardiotocography (CTG, high risk pregnancies in obstetrician-led care). Severe CTG abnormalities are used in the modified ReCoDe as a proxy for foetal hypoxia [21].

The main outcomes of a local perinatal audit concerns SSFs and recommendations. In each discussed case, all possible SSFs are collected by the local perinatal audit team, entered in PARS and used to change practice in order to prevent future SSFs of the same type. At the next local perinatal audit, the SSFs from the previous audit are presented to all present obstetric caregivers. The aim of this process is to improve the quality of care. For each perinatal death, multiple SSFs could be entered in free description fields. An SSF was defined as “a care management problem involving care that deviated from the safe limits of practice as laid down in guidelines, standards, protocols or normal practice” [23–25]. The category ‘guidelines’ is used to describe deviations from care as laid down in documents on practice/management by obstetric caregivers. The category ‘usual care’ is used for what is considered as standard acts of care by professional caregivers. The subclassifications of ‘usual care’ for this study were based on examples from Eskes et al. and from a national report on term perinatal mortality audit [9, 23]. The probability that the SSF was related to the cause of death was subdivided into: *very likely*, *likely*, *possible*, *indeterminable*, *unlikely*, *none*, and *no consensus*. The cases in the groups *very likely*, *likely* and *possible* were analysed to examine the SSF’s relation to the cause of death. In line with the INDEXstudy, we selected data of the SSFs in perinatal deaths beyond 41 weeks of gestation. For this reason, we did not make a comparison with SSFs in the early−/full-term pregnancy period.

### Statistics

Descriptive statistics were used to describe differences between early−/full-term and the late−/postterm group and to describe SSFs in perinatal deaths in pregnancies of 41 weeks and beyond. No statistical analyses were performed, due to the rare occurrence of the outcome, the content of data and the design of the study.

## Results

**Fig. 1:**
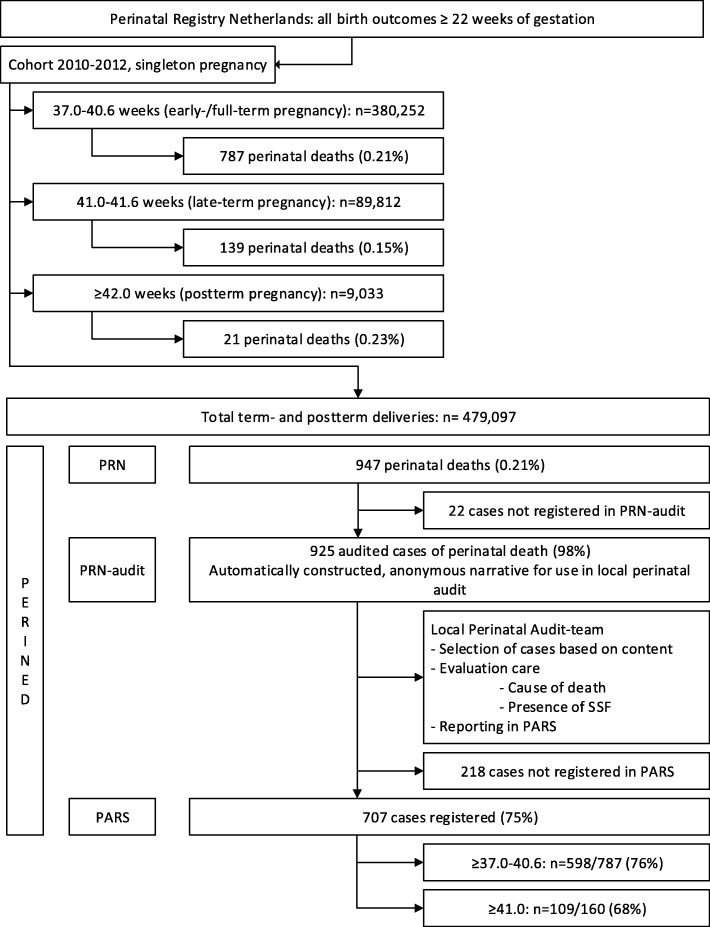
Flowchart of selection of cases of perinatal death ≥37 weeks between 2010 and 2012 Perinatal Registry Netherlands 2010–2012: all birth outcomes in pregnancies ≥22 weeks of gestation

Between 2010 and 2012, 947 perinatal deaths in pregnancies of ≥37.0 were registered in the PARS database. From these registered deaths, 925 (98%) were audited and 707 (75%) were registered in PARS, from which 8 with an ‘unknown’ timing of death [9, 26–28]. No extra information could be obtained from the 218 (24%) perinatal deaths that were not registered in PARS (Fig. 1). Based on the Wigglesworth/Hey classification (WHEN), it was possible to stratify 598/787 (76%) registered perinatal deaths to the early−/full-term group (37.0–40.6) and 109/153 (71%) to the late−/postterm group (≥41.0) (Fig. 1). Stratified by moment of death we could identify 440 stillbirths and 259 neonatal deaths (Table 1). Antepartum stillbirth was registered in 55.2% in the early−/full-term group and in 42.2% in the late−/postterm group. Intrapartum stillbirth was registered 7.2% in the early−/full-term group and in 19.3% the late−/postterm group.

**Table 1:** Term stillbirth and neonatal death stratified by gestational age registered in the Dutch Perinatal Audit system

Gestational age (wk)	Stillbirth (*n* = 440)				Neonatal death (*n* = 259)				Missing		Total
	Antepartum		Intrapartum		Early (day 0–6)		Late (day 7–27)				
≥ 37.0–40.6 (n, %)	330	55.2%	43	7.2%	166	27.8%	51	8.5%	8	1.3%	598
≥ 41.0 (n, %)	46	42.2%	21	19.3%	31	28.4%	11	10.1%			109
Total (n, %)	376	53.2%	64	9.1%	197	27.9%	62	8.8%	8	1.1%	707

See Fig. 1 for national cases of term perinatal death between 2010 and 2012

In Table 2, the most common classification of perinatal death (Modified ReCoDe) is shown, as determined by the local perinatal audit, stratified by gestational age and subdivided by congenital anomalies. Foetal congenital anomalies were found in 15% in the early−/full-term group and in 4% in the late−/postterm group. In foetus/neonates without congenital anomalies, foetal asphyxia occurred in 19% of the early−/full-term group in comparison to 33% in the late−/postterm group. Intrapartum asphyxia occurred in 16% in the early−/full-term group and 34% in the late−/postterm group (Table 2). Acute infections, neonatal asphyxia and BMI ≥25 kg/m^2^ are less often classified as reason of death in the early−/full-term group in comparison to the late−/postterm group. In 16 cases, both foetal asphyxia and intrapartum asphyxia were selected by the local perinatal audit in the same case.

**Table 2:** Most common classification of perinatal death (Modified ReCoDe) stratified by gestational age

Classification	Subclassification	37.0–40.6 weeks (ref)		≥ 41 weeks	
		N	%	N	%
Overall		598		109	
Foetal/antepartum	Congenital anomaly	89	15%	4	4%
Neonatal	Congenital anomaly	104	17%	8	7%
Without congenital anomalies		441		97	
Foetal/antepartum	Infection: acute	13	3%	8	8%
	Asphyxia^a^	82	19%	32	33%
Neonatal	Asphyxia	63	14%	20	21%
Maternal	Risk factor: overweight (BMI ≥ 25)	100	23%	33	34%
Intrapartum	Asphyxia^a^	70	16%	33	34%

^a^16 cases of perinatal death are registered in both foetal asphyxia and in intrapartum asphyxia

For each case ≥1 condition could be entered

**Fig. 2:**
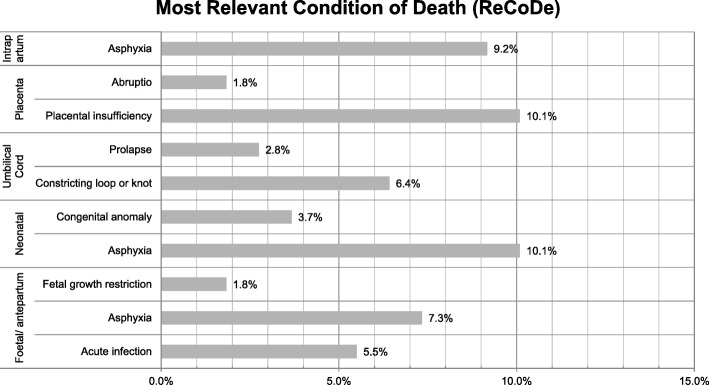
Most relevant condition of death in 109 cases of perinatal death in late−/postterm pregnancy with an incidence of > 1%

The most frequent, most relevant condition causing death in the full late−/postterm group according to the local perinatal audit was foetal asphyxia in 63/109 (57.8%), intrapartum asphyxia in 9.2% (10/109), neonatal asphyxia in 10.1% (11/109) and placental insufficiency in 10.1% (11/109) (Fig. 2).

A total of 178 SSFs were identified in 109 cases of perinatal death in pregnancies of 41 weeks and beyond. In 75 cases (68.8%), at least one or more SSFs could be identified (Table 3). There were no differences in number of cases with SSFs across 2010–2012. In 63/178 (35.4%) cases, SSFs were identified as a *very likely*, *likely* and *possible* relation to the cause of death. The most frequent reported SSFs in this group concerned ‘CTG evaluation and classification’ in 11/109 (10.1%), ‘CTG registration or documentation’ in 5/109 (4.6%) and ‘inadequate management in reduced foetal movements’ in 5/109 (4.6%).

**Table 3:** Number and content of substandard care factors (SSF) per perinatal death case ≥41 weeks as identified by the local perinatal audit

	n	%
Cases of perinatal death	109	
≥ 1 SSF	75	68.8%
1 SSF	29	38.7%
2 SSF	14	18.7%
3 SSF	17	22.7%
≥ 4 SSF (max 8)	15	20.0%
No SSF	27	24.8%
Missing/insufficient data	7	6.4%
SSF with possible/ likely/ very likely relation to death^a^	63	57.8%
Guideline- oriented SSF		
Cardiotocography evaluation or classification	11	10.1%
Cardiotocography registration or documentation	5	4.6%
Guideline obesity	3	2.8%
Usual Care- oriented SSF		
Midwifery guidelines	4	3.7%
Documentation in the medical records	3	2.8%
Decreased foetal movements	5	4.6%
Patient factor	3	2.8%
Total number SSF	178	

^a^Unknown/unclear cases are not presented

## Discussion

### Main findings

Perinatal death in term- and postterm pregnancy is rare. In our study, stillbirth occurred less often antepartum and more often intrapartum in pregnancies ≥41.0 weeks in comparison to pregnancies at 37.0–40.6 weeks. Asphyxia was seen more frequently as the most relevant condition causing death in pregnancies ≥41.0 weeks. Substandard factors with a likely relation to death in pregnancies ≥41.0 weeks concerned inadequate CTG monitoring (evaluation, classification, registration or documentation) in 14.7% (16/109) and ‘reduced foetal movements in 4.6%.

### Validity of the results

This nationwide study shows an audited cohort regarding the perinatal mortality in the Netherlands. Of the late−/postterm perinatal deaths, 68% (109/160) were registered in PARS, which is comparable to the 76% (598/787) of the early−/full-term registered perinatal deaths [26–28]. It was described in the study of Eskes et al. that —although not all term cases of perinatal death are audited—characteristics of the audited cases like parity, maternal age, and gestational age, are comparable with all term perinatal deaths in the national registration of the Netherlands Perinatal Registry (PRN) [23]. We assume that this statement also applies on our cohort, since we have used the same source of data, though comparisons with other studies should be made keeping this selection of cases in mind.

Audit teams auditing perinatal care is becoming more and more part of common care, with the aim to reduce perinatal mortality and improve the quality of care at all levels of the health system [29–32]. Efforts to prevent perinatal and maternal deaths will improve perinatal and maternal outcomes, which are markers of the quality of care in pregnancy and childbirth, also in high-income countries [33, 34].

Eskes et al. assessed the implementation and the results of the Perinatal Audit in 2010–2012 of pregnancies ≥37 weeks in the Netherlands using PARS data. They showed that total stillbirth in the early−/full-term period was comparable to the late−/postterm period, without making the sub-classification in ante- and intrapartum stillbirth like shown in our study [23].

There are different ways to describe perinatal and maternal death [35]. Perined uses Wigglesworth/Hey and the modified ReCoDe to determine timing and cause of death [21]. Internationally, the ICD-PM is often used to describe deaths during the perinatal period and is based on the coding rules of the ICD-10 (International Statistical Classification of Diseases and Related Health Problems) and allows comparisons of perinatal death between settings) [36, 37]. The ICD-PM first codes the timing of death (antepartum, intrapartum or neonatal) and is comparable to the Wigglesworth/Hey. Secondly, ICD-PM assigns the main cause of perinatal death, which is comparable to the modified ReCoDe we used. Lastly, ICD-PM assigns the main maternal condition at the time of perinatal death, which is also part of the classification by Perined. Although both classification systems are broadly comparable, Perined should be advised to introduce ICD-PM for future studies and to be able to compare with international data.

When using term singleton deliveries registered in the PRN, the proportion of perinatal death is lowest (0.15%) in the late-term pregnancy period (41.0–41.6), 0.21% in the early−/full term period and 0.23% in the postterm pregnancy period. In the PRN data, the proportion of foetal death (0.14% vs 0.11%) and neonatal death (0.07% vs 0.06%) is slightly higher in the early−/full-term group in comparison to the late- and postterm group. When using the selected cases of perinatal deaths from the PARS database, the proportion of stillbirth exceeds the proportion of neonatal mortality in both the early−/full-term group and the late−/postterm group. The systematic review of Gulmezoglu on induction of labour in women at or beyond term, showed no differences in timing of induction regarding perinatal death [6]. Our results are in concordance with the observations of Mandujano et al., who analysed more than 8 million pregnancies in 2003–2005 in the US, showing that at 37–38 weeks of gestation the number of neonatal deaths is lower than the level of stillbirths [38]. Our results are in contrast with the results of Rosenstein et al., who analysed nearly 4 million pregnancies in 1997–2006 in the state of California, showing that the proportion of infant death (defined as number of children who died in the first year of live) exceeds the proportion of stillbirths in the term period in all ethnicities [39]. This difference could be due to a pregnancy dating based on last menstrual period instead of early ultrasound and we were only able to analyse data on perinatal death during the first month instead of the first year. In a Swedish cohort study from 1982 to 1991, the stillbirth rate was highest for primiparas at 38 completed weeks (0.27%), lowest at 40 weeks (0.12%), and 0.23% in the postterm period [8]. All mentioned studies concern other methods of pregnancy dating and other timeframes than our study which makes a clear comparison difficult.

In our Perinatal Audit study, foetal and intrapartum asphyxia was identified more in the late−/postterm death group than in the early−/full-term group in the present study. In 16 cases, both foetal and intrapartum asphyxia was selected in the ReCoDe. In some cases, it could be hard to differentiate whether the pathological process of asphyxia started before or during labour. If antepartum asphyxia occurred, this process would most probably continue throughout labour, which could have contributed to the double selection in ReCoDe. Hereby, we could have both over- and underestimated the proportion of foetal and intrapartum asphyxia. The placental function is presumably the most important factor contributing to asphyxia in general and to asphyxia in foetal growth restriction [40, 41]. Regarding intrapartum asphyxia, Berglund et al. reported a threefold increased risk for asphyxia in postterm deliveries compared to term deliveries [42]. In postterm pregnancy, labour is induced and monitored by CTGevaluation, which is the most important SSF in our audit reports. When labour is induced, there is a risk of hyperstimulation which, if not recognised, could contribute to asphyxia. In late−/postterm pregnancy, one of the most important risk factors associated with increased perinatal mortality is foetal growth restriction [43, 44] However, in the present study foetal growth restriction was classified as most important reason of death in 1.8% and classified as ‘relevant’in 6.4%. This difference in incidence could be due to the way data was entered: Asphyxia is considered a more contributing factor to perinatal death than growth restriction resulting in a higher registration of this risk factor. Also, at suspected growth restriction, most women are induced at 40 weeks, resulting in a lower overall incidence [44].

There is no uniform international standardisation of SSFs categories and definitions, making it difficult to compare the results of studies with each other and with our present study. The possibility to compare with other studies would help us to learn from SSFs demonstrated by others, keeping in mind the level of development of obstetric care in each country. In the EuroNatal study, 1619 cases of perinatal death ≥28 weeks were analysed in an external audit. SSFs with a possible or probable relation to death were found in 46% of the cases. [15] In 63/109 (57.8%) of the cases in this study an SSF with a *very likely*, *likely* or *possible* relation to death was found. According to the EuroNatal results, the most important SSF was undetected foetal growth restriction and in 10% it was seen as a cause that might have or was likely to have contributed to death [15].

In our study, the main reason for an SSF with a relation to death, were inadequate CTG evaluation or classification in 11/109 (10.1%) and registration or documentation in 5/109 (4.6%). Berglund et al. performed a case-control study in which neonates with and without an Apgar score of < 7 at 5 min were compared regarding substandard care during labour. The main finding of substandard care was “misinterpretation of CTG, not acting on an abnormal CTG in a timely fashion and incautious use of oxytocin” [45]. In an earlier study of Berglund et al., staff did not act timely on pathological CTGs in 71% of the pregnancies [46]. In a study by Batlle et al., in which the quality of intrapartum care regarding birth asphyxia was assessed, two of the main shortcomings were related to misinterpretation of CTG and a delayed response time to CTG anomalies [47]. Auditing cases of perinatal death/asphyxia often classifies a CTG as non-reassuring in retrospect, while it is known that there is a poor inter-observer agreement on classification and management on non-reassuring CTGs [48]. These studies, as well as the present study, show the importance of adequate execution of CTG monitoring to reduce substandard care and to improve birth outcome.

### Strengths and limitations

The perinatal audit was not primarily set up for scientific purposes, but as an instrument to monitor and improve the quality of perinatal healthcare. Because PARS is set up as an anonymous database it was not possible to link the data to the national perinatal registry or check the source files, resulting in missing maternal and perinatal information on case level. Especially the impossibility to distinguish between late-term and postterm pregnancy, parity, level of care during pregnancy and delivery, onset of labour (spontaneous onset or labour induction) and mode of delivery are major limitations of the current PARS registry. This makes it difficult to draw conclusions on the level of obstetric management and is the main reason we did not perform statistical analyses [9, 13, 23]. Another limitation is that only 75% of all cases are audited and entered in PARS. Cases with a clear cause of death are sometimes not audited, which results in a selected group of perinatal deaths registered in PARS and could contribute to an over−/underestimation in timing of perinatal death, causes of perinatal death and SSFs. Though auditing cases of maternal/perinatal/adverse events is getting more and more ‘standard care’, it is still not obligatory to perform an audit, or to enter audited cases in the PARS database. This reflection on management of care could be a quality requirement to ensure high standards in obstetric care in the Netherlands.

A limitation of the SSF classification system is that this is not standardized, which makes it more difficult to compare our results to other countries. The main outcome regarding inadequate CTG monitoring could not be differentiated into monitoring prior or during delivery.

### Recommendations

There is an urgent need for a uniform international classification system of SSFs. The Groningen system has a moderate to good inter-rater agreement on well-defined (sub) categories, with clear guidelines and examples, which can be used to standardise the SSFs in the PARS database together with the studies of Eskes [49]. In addition, the PARS database should also make some basic characteristics and obstetric characteristics (eg spontaneous onset of labour/induction) obliguatory to fill in and use the ICD-PM classification in order to make the database more suitable for future evaluation. Finally, in pregnancies ≥41 weeks, care providers should be aware of the risk of intrapartum asphyxia and in those pregnancies where CTG is indicated, attention should be paid to adequate CTG registration, evaluation and classification.

## Conclusions

Pregnancies with a gestational age at or beyond 41 weeks, from which 75% registered in the Dutch Perinatal Audit system, showed less antepartum stillbirth and more intrapartum stillbirth than pregnancies of 37.0–40.6 weeks. More foetal, intrapartum, and neonatal asphyxia were identified as causes of death in pregnancies beyond 41 weeks compared to pregnancies of 37.0–40.6 weeks. The most identified SSFs with a relation to death in pregnancies beyond 41 weeks concerned inadequate CTG monitoring (evaluation, classification, registration or documentation) and ‘inadequate action on decreased foetal movements’.

## Abbreviations

CTG: Cardiotocography
PAN: Perinatal Audit in the Netherlands (now Perined)
PARS: Perinatal Audit Registry System
PRN: Perinatal Registry Netherlands (now Perined)
ReCoDe: Relevant Condition of Death
SSFs: Substandard care factors
